# Correlation Constraints for Regression Models: Controlling Bias in Brain Age Prediction

**DOI:** 10.3389/fpsyt.2021.615754

**Published:** 2021-02-18

**Authors:** Matthias S. Treder, Jonathan P. Shock, Dan J. Stein, Stéfan du Plessis, Soraya Seedat, Kamen A. Tsvetanov

**Affiliations:** ^1^School of Computer Science & Informatics, Cardiff University, Cardiff, United Kingdom; ^2^Department of Mathematics and Applied Mathematics, University of Cape Town, Cape Town, South Africa; ^3^National Institute for Theoretical Physics, Matieland, South Africa; ^4^SA MRC Unit on Risk & Resilience in Mental Disorders, Department of Psychiatry and Neuroscience Institute, University of Cape Town, Cape Town, South Africa; ^5^Department of Psychiatry, Faculty of Medicine and Health Sciences, Stellenbosch University, Cape Town, South Africa; ^6^Department of Clinical Neurosciences, University of Cambridge, Cambridge, United Kingdom; ^7^Department of Psychology, University of Cambridge, Cambridge, United Kingdom

**Keywords:** age, brain, optimization, prediction, correlation, regression

## Abstract

In neuroimaging, the difference between chronological age and predicted brain age, also known as *brain age delta*, has been proposed as a pathology marker linked to a range of phenotypes. Brain age delta is estimated using regression, which involves a frequently observed bias due to a negative correlation between chronological age and brain age delta. In brain age prediction models, this correlation can manifest as an overprediction of the age of young brains and an underprediction for elderly ones. We show that this bias can be controlled for by adding correlation constraints to the model training procedure. We develop an analytical solution to this constrained optimization problem for Linear, Ridge, and Kernel Ridge regression. The solution is optimal in the least-squares sense i.e., there is no other model that satisfies the correlation constraints and has a better fit. Analyses on the PAC2019 competition data demonstrate that this approach produces optimal unbiased predictive models with a number of advantages over existing approaches. Finally, we introduce regression toolboxes for Python and MATLAB that implement our algorithm.

## 1. Introduction

As the world's population ages, early detection and prevention of neurological aspects of aging, such as cognitive decline and dementia, is a public health priority and challenge. Pathological aging could be indicated by the level of deviation from the typical pattern of aging in healthy individuals (1). There has been growing interest in developing statistical approaches in order to identify individuals deviating from a healthy brain aging trajectory (2). To this end, a metric referred to as *brain age delta*, defined as the difference between brain-predicted age and chronological age, has been proposed as an index of the level of neuropathology in aging (2–4). Investigating the association between this metric with demographics, and lifestyle and cognitive variables can deepen the understanding of the processes that underpin healthy aging (5). In clinical research, brain age delta has the potential to index the severity of premature aging in patients suffering from disease. Among others, a higher delta has been associated with lower fluid intelligence and higher mortality (1), risk for developing Alzheimer's disease (6), severity of schizophrenia and depression (7).

Establishing a good estimate of brain age delta is faced with important methodological challenges. The first challenge relates to the kinds of features (biomarkers) and predictive models that are used to build a brain-age model. A number of brain metrics have been considered as features for regression models; for example, structural networks (8), cortical thickness (9), functional connectivity patterns (10, 11), and raw T1-weighted images (1, 2). Likewise, a variety of regression models has been tested, from linear regression models such as lasso and support vector regression (10, 11) to convolutional neural networks [CNNs; (2, 8)]. The quest for more accurate brain-age models lies at the heart of the Predictive Analytics Competition (PAC) 2019 upon which the eponymous Frontiers Research Topic is founded^1^.

A more fundamental methodological challenge, and the starting point for this paper, is the very operationalization of the brain age delta. If we denote the chronological age for a set of participants as a vector **y**, the ages predicted on the brain scans as $\hat{\text{y}}$, and the residuals as $\text{e}=\text{y}-\hat{\text{y}}$, then the negative residual δ = −**e** (i.e., predicted brain age minus chronological age) is usually defined as the brain age delta. This metric has been shown to be problematic. A predictive bias manifesting as an overprediction of the age of young individuals and an underprediction for elderly individuals has led to much speculation and investigation (2, 3, 9, 12–15).

**Figure 1:**
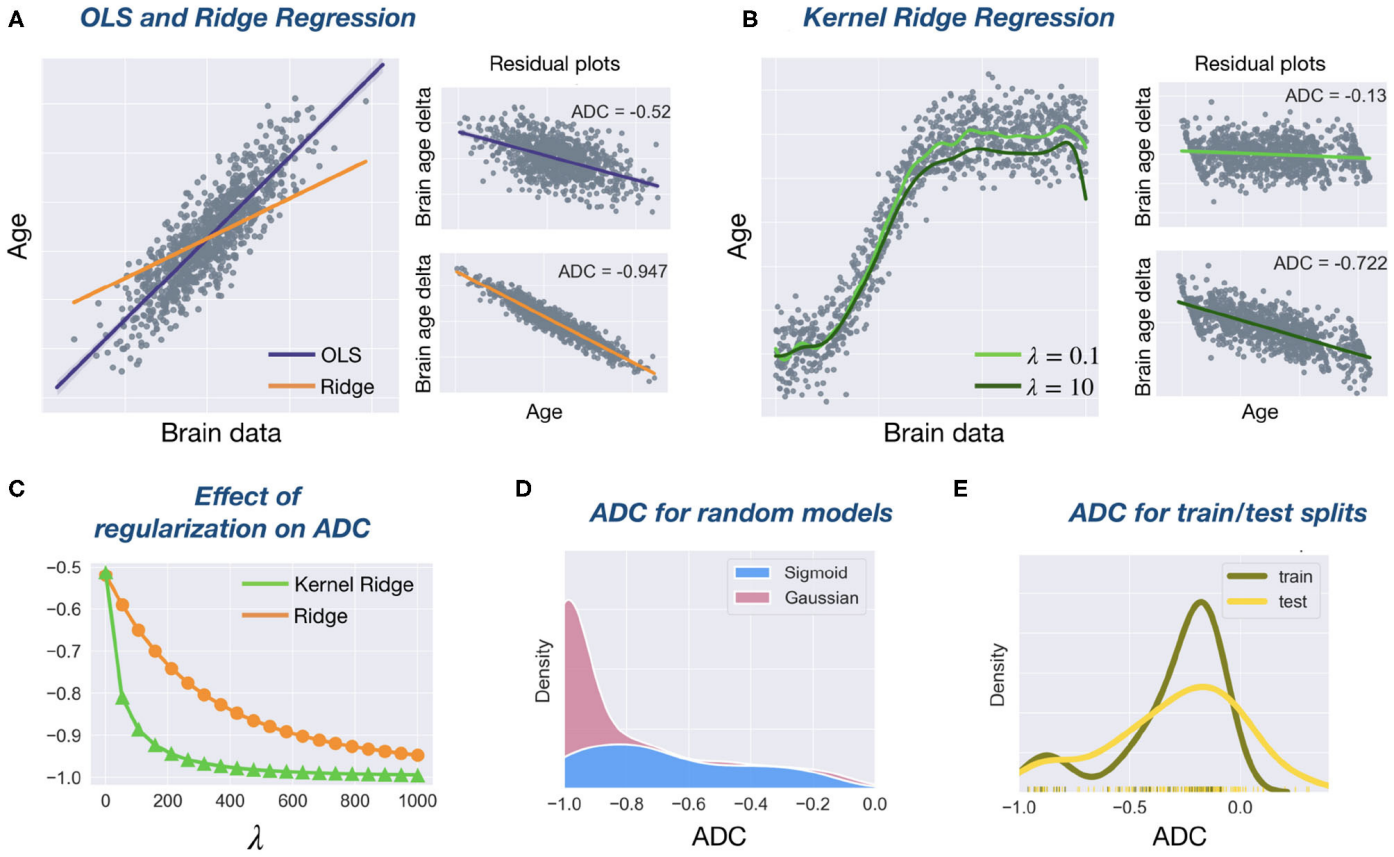
Investigating age delta correlation (ADC) with simulated data. **(A)** Simulated data (1,000 samples, 1 feature, linear function, Gaussian noise) using Scikit-Learn's make_regression function. Regression line fits for OLS and Ridge (λ = 1, 000) regression are shown. The small residual plots (plotting y vs. brain age delta) show a significant negative ADC for both models. **(B)** Simulated data (1,000 samples, 1 feature, sigmoid function, Gaussian noise) with two Kernel Ridge regression fits (RBF kernel, *γ* = 1) for two different regularization strengths λ. Again, residual plots show a high ADC. **(C)** For both Ridge and Kernel Ridge regression, increasing the regularization hyperparameter λ leads to more negative ADC. This effect is more pronounced for Kernel Ridge than Ridge. **(D)** ADC density plot. We sampled 100 random Gaussian and sigmoid datasets as specified before. We created random regression models by sampling slopes and intercepts from a uniform distribution (the sampling space included the OLS coefficients). When calculating ADCs on random models, a clear bias toward large negative ADC values is evident. Its distribution is more peaked for the Gaussian data. **(E)** ADC density plot. Simulated data (1,000 samples, 5 features, linear function, Gaussian noise) is split into 50% train, 50% test data. An OLS model is calculated on the train data and ADC is calculated on both train and test data. For the test data, the variability of the ADC is larger than for train data, and its mode is slightly more negative.

A useful quantification of this effect is the correlation between chronological age and delta, corr(**y**, **δ**), which we will refer to as *age delta correlation (ADC)* in the rest of the paper (see Figures 1A,B). An analysis by (15) showed that negative ADC is ubiquitous across a range of aging datasets and regression models and independent of the age range included in the dataset. A theoretical analysis by (14) showed that this effect is an inevitable property of regression, further aggravated by regression dilution (16, 17), and hence not limited to aging data. The potential danger of non-zero ADC lies in spurious associations with other covariates: brain age delta can be trivially correlated with demographic or cognitive variables if the latter are correlated with chronological age as well. Le et al. (14) found that associations between residuals and variables obtained from clinical interviews and neuropsychological testing largely disappear when residuals are corrected for chronological age. To avoid these spurious correlations, several authors have suggested following up the regression analysis with a correction step wherein the effect of age is removed from the residuals (1, 3, 12, 14). Brain age delta is then calculated by the following two-stage approach:

1. *Brain Age Prediction*. Train a regression model to predict age from neuroimaging data. The difference between predicted age and chronological age reflects *uncorrected brain age delta*.
2. *Correction of Brain Age Delta*. Use simple linear regression to regress delta against age. The resultant residuals are uncorrelated with age and denoted as *corrected brain age delta*.

Despite the significant methodological progress that has been made there are still concerns that warrant attention. First, the correction approach is an *ad hoc* fix because the models' predictions do not take ADC into account. Second, in a strictly sequential two-stage approach it is not clear whether the resultant brain age delta is optimal in terms of predictive accuracy. Both issues can be addressed when prediction and correction are unified within a model. Third, in predictive settings with training and test set, zeroing ADC on the training set is not of primary importance. Rather, it would be useful to have a model that is able to finely control the trade-off between ADC and predictive accuracy in order to optimize its performance on the test set.

The aim of this study was to address these points by introducing modifications to three different regression models (Linear, Ridge and Kernel Ridge regression). Our models explicitly control for ADC on the training set without the need for an additional correction after model training. This was realized by formulating constrained optimization problems that incorporate age delta correlation as additional constraints. Our approach offers the following features:

- *Predictive Model*. In predictive modeling, all properties of the estimation pipeline (including correction of the residuals) should be derived from the training set and validated on a separate test set. Some of the existing approaches conflate training and test data because age prediction is based on the training data but the correction is performed on the test set. The latter introduces dependencies between test samples because the correction applied to a test sample depends on the other test samples.
- *Arbitrary Test Set Size*. An additional problem arising from conflating training and test sets is that performing correction on test data requires a sufficiently large test set. This is especially problematic with smaller datasets because less data is available for training. Since our approach estimates all parameters from the training data, it can be applied with any train/test split including leave-one-out cross-validation, when appropriate.
- *Prediction of Unlabeled Data*. Our model corrects the predictions not the residuals. Therefore, corrected predictions can be obtained even on unlabeled data if necessary.
- *Optimality*. Formulating both model training and correction as a single constrained optimization problem allowed us to show that the resultant models are optimal in terms of mean-squared error (MSE) on the training set. In other words, of all potential solutions that control ADC, our solution has the highest accuracy.
- *Correlation Bound*. Our models allow for “soft” control of the ADC by defining a correlation bound that caps the maximum permissible correlation, e.g., |corr(**y**, **δ**)| ≤ 0.1. This is especially useful for predictive modeling, because minimizing ADC on the training set is not vital. Rather, a low bias and good predictive performance on the test set is desired. Using a correlation bound, our models allow for fine-tuning of the trade-off between ADC and predictive accuracy.
- *Interpretability*. An advantage of unifying prediction and correction in a single model is better interpretability because the entire operation of the model is represented by its regression coefficients and quantities derived from them (18–20). Furthermore, we show in Section 2.7 that these quantities are not affected by the choice of the correlation bound (a hyperparameter in our model).

Some of the existing approaches share some of the listed features. For instance, whereas in (14) test set residuals are used for correction, Beheshti et al. (12) learns the correction parameters from the training set and applies them to the test set, in line with good practice for predictive models. However, to the best of our knowledge, this is the first study to prove optimality of the models and introduce “soft” correlation bounds for fine control of ADC.

## 2. Method

The section 2 is organized as follows. In Section 2.2, we introduce Linear regression, Ridge regression and Kernel Ridge regression. In Section 2.3, we review existing ways to quantify brain age delta. In Section 2.4, we revisit the mathematical basis for ADC in the context of the three regression models. In Section 2.5 we develop our approach by adding correlation constraints to the model training stage that allow for a precise control of ADC. In Section 2.6 it is shown that the brain age estimates obtained with our models are closely related to existing correction approaches. In Section 2.7, we investigate how to interpret the models. In Section 2.8, we introduce corresponding toolboxes for Python and MATLAB that implement them. Finally, in sections 2.9 and 2.10, we describe our analysis of the PAC2019 competition data.

### 2.1. Notation

Table 1 defines the most important mathematical symbols used in the paper. Whenever the symbol represents a vector or matrix, its dimensionality is given in the second column. In general, matrices are denoted as uppercase boldface symbols (e.g., **X**, **H**), vectors as lowercase boldface symbols (e.g., ***β***, $\bar{\text{x}}$), and scalars as lowercase normal face symbols (e.g., *n*, ȳ). Note that we use terminology common in the machine learning literature. In particular, *features* are also known as predictors or independent variables in the regression literature, the vector of *targets* (chronological age) is also known as response vector or dependent variable, and *training* is also known as fitting. For standard regression models, regression coefficients are denoted as ***β***. If an intercept is included in the model, we assume that a column of 1's is added to the matrix of features **X**. Sometimes we explicitly denote the intercept as ***β***_0_ and the non-intercept coefficients as ***β***_1:*p*_. For models with correlation constraints, we use the notation ***b*** for the regression coefficients with ***b***_0_ and ***b***_1:*p*_ defined analogously.

**Table 1:** Mathematical notation, dimensionality, and description for the main quantities used in the regression models.

*n*	ℕ	Number of samples
*p*	ℕ	Number of features
***β***	ℝ^*p*+1^	Coefficients for standard regression model
***b***	ℝ^*p*+1^	Coefficients for correlation constrained model
**X**	ℝ^*n* × *p*^	Brain scans (features)
**y**	ℝ^*n*^	Chronological age (targets)
**$\hat{\text{y}}$**	ℝ^*n*^	Predicted age (**$\hat{\text{y}}=\text{X}\beta$**)
**e**	ℝ^*n*^	Residuals (**$\text{e}=\text{y}-\hat{\text{y}}$**)
δ	ℝ^*n*^	Uncorrected brain-age delta (negative residuals *δ* = −**e**)
*ρ*	ℝ	Correlation bound (hyperparameter)
⊤		Transpose operator
**1**	ℝ^*n*^	Vector of 1's
**$\bar{\text{x}}$**	ℝ^*p*^	Column means of **X** given by $\frac{1}{n}{\text{x}}^{⊤}1$
*ȳ*	ℝ	Mean of **y**

### 2.2. Regression Models

#### 2.2.1. Ordinary Least-Squares (OLS) Regression

Ordinary least-squares regression, often just called linear regression, can be formulated as a set of *n* equations of the form

$$y_{i}=\beta_{0}+\beta_{1}x_{i1}+\beta_{2}x_{i2}+\ldots +\beta_{p}x_{ip}+\epsilon_{i}\;i=1,2,\ldots ,n$$

where *y*_*i*_ is the *i*-th response, *x*_*ij*_ is the *j* − *th* predictor value in the *i*-th sample, the *β*’s are regression coefficients, and $\epsilon \;\sim \;N\left(0,\sigma^{2}\right)$ is an error term. Using matrix notation, this set of equations can be written more succinctly as:

(1) $$\begin{array}{l} \text{y}=\text{X}\beta +\epsilon \end{array}$$

where $\text{y}={\left[y_{1},y_{2},...,y_{n}\right]}^{⊤}$ comprises the responses, **X** ∈ ℝ^*n* × (*p*+1)^ is the matrix of features including a column of 1's for the intercept term, ***β*** ∈ ℝ^*p*+1^ is the vector of regression coefficients and $\epsilon ={\left[\epsilon_{1},\epsilon_{2},...,\epsilon_{n}\right]}^{⊤}$ collects all error terms. Training a model implies finding an estimate for ***β*** such that **y** ≈ **X*****β***. In OLS regression this is achieved by minimizing the sum of squared errors $\|\text{y}-\text{X}\beta {\|}_{2}^{2}$. Denoting $\hat{\text{y}}:=\text{X}\beta$ this can be formulated as the unconstrained optimization problem

(2) $$\begin{array}{l} \text{minimize }\|\text{y}-\hat{\text{y}}{\|}_{2}^{2}. \end{array}$$

The solution is given by

$$\begin{array}{l} \beta_{\text{ols}}={\left({\text{X}}^{⊤}\text{X}\right)}^{-1}{\text{X}}^{⊤}\text{y}. \end{array}$$

#### 2.2.2. Ridge Regression

OLS regression suffers from high variance and does not have a unique solution if the number of the features *p* is larger than the number of samples *n* (21, 22). Ridge regression is a regularized version of OLS regression that is useful for data that suffers from multicollinearity. The model is regularized by adding an ℓ_2_ penalty that shrinks the weights toward zero. For a given regularization parameter λ ≥ 0, Ridge regression can be formulated as the unconstrained optimization problem

$$\begin{array}{l} \text{minimize }\|\text{y}-\hat{\text{y}}{\|}_{2}^{2}+\lambda \|\beta_{1:p}{\|}_{2}^{2}. \end{array}$$

The first term is the least-squares term from Equation (2). The second term penalizes elements of ***β*** from becoming too large. For λ = 0 Ridge regression reduces to OLS regression. The solution is given by

(3) $$\begin{array}{l} \beta_{\text{ridge}}=\;{\left({\text{X}}^{⊤}\text{X}+\lambda \text{I}\right)}^{-1}\;{\text{X}}^{⊤}\text{y} \end{array}$$

where **I** ∈ ℝ^(*p*+1) × (*p*+1)^ is an identity matrix. Since the intercept term is not regularized, **I** is modified such that the 1 in the first row/column is replaced by a 0.

#### 2.2.3. Other Linear Regression Models

Other variants of linear regression e.g., lasso (23) and elastic net (24), do not have a closed form solution but rely on iterative optimization, so they do not lend themselves to the analytical approach developed in this paper.

#### 2.2.4. Kernel Ridge Regression (KRR)

A non-linear version of Ridge regression can be developed by applying a non-linear transformation to the features and then performing Ridge regression on these transformed features (25). Let this transformation be represented by a map $\phi :{\mathbb{R}}^{p}\to F$ from input space to a higher-dimensional Reproducing Kernel Hilbert Space and $\Phi \left(\text{X}\right)={\left[\phi \left({\text{x}}_{1}\right),\phi \left({\text{x}}_{2}\right),...,\phi \left({\text{x}}_{n}\right)\right]}^{⊤}$ (26, 27). The solution is given by replacing **X** by Φ(**X**) in Equation (3),

$$\begin{array}{l} \beta_{\text{krr}}=\;{\left(\Phi {\left(\text{X}\right)}^{⊤}\Phi \left(\text{X}\right)+\lambda \text{I}\right)}^{-1}\;\Phi {\left(\text{X}\right)}^{⊤}\text{y}. \end{array}$$

This solution, also known as primal solution, is of limited practical use, since the feature space is often too high-dimensional to represent ***β***_krr_ and Φ(**X**). As an alternative, the convex optimization problem can be rewritten into its dual Lagrangian form first (28). The resultant dual solution is given by

(4) $$\begin{array}{l} \beta_{\text{krr}}=\;\Phi {\left(\text{X}\right)}^{⊤}{\left(\Phi \left(\text{X}\right)\Phi {\left(\text{X}\right)}^{⊤}+\lambda \text{I}\right)}^{-1}\;\text{y}. \end{array}$$

The equivalence between the primal and dual solution can be verified by left-multiplying both solutions with (Φ(**X**)Φ(**X**)^⊤^+ λ**I**). Since ***β***_krr_ cannot be represented directly, we instead calculate a vector of dual weights **α** ∈ ℝ^*n*^. To this end, define **K** = Φ (**X**) Φ (**X**)^⊤^ as the kernel matrix with **K**_*ij*_ = *k*(**x**_*i*_, **x**_*j*_) for a kernel function *k*. Then the vector of dual weights is given by the latter part of Equation (4),

(5) $$\begin{array}{l} \alpha ={\left(\text{K}+\lambda \text{I}\right)}^{-1}\;\text{y}. \end{array}$$

Using **α** the predicted response to a test sample **x** can be rewritten in terms of kernel evaluations:

(6) $$\begin{array}{l} f\left(\text{x}\right)=\beta_{\phi }^{⊤}\phi \left(\text{x}\right)=\alpha^{⊤}\Phi \left(\text{X}\right)\phi \left(\text{x}\right)=\overset{n}{\underset{i=1}{\sum }}\alpha_{i}k\left({\text{x}}_{i},\text{x}\right). \end{array}$$

### 2.3. Calculating the Brain-Age Delta

The regression of age on brain features often leads to a biased model that manifests as an overprediction of the age of younger individuals and an underprediction of the age of elderly ones. This effect can be quantified as a negative age delta correlation (ADC) denoted as corr(**y**, ***δ***). In the literature, ADC has been set to zero by adding a second stage to the analysis wherein the regression predictions from the first stage are corrected. Hence, brain-age delta prediction can be formulated as the following two-stage approach:

(a) *Brain Age Prediction*. Train a regression model *f* to predict age such that **y** ≈ *f* (**X**). The negative residuals, denoted as
  (7) $$\begin{array}{l} \delta =f\left(\text{X}\right)-\text{y} \end{array}$$
represent the uncorrected brain age delta.
(b) *Correction of Brain Age Delta*. A number of authors proposed correction procedures to rid ***δ*** of ADC (1, 3, 4, 12, 14). Many of these approaches are mathematically equivalent. They boil down to two approaches that yield two differently corrected residuals ***δ***_1_ (approach 1) and ***δ***_2_ (approach 2). These two approaches are discussed in detail in the following two subsections.

#### 2.3.1. Approach 1: Scale Down **y** (Chronological age)

Approach 1 has been proposed by a number of authors (3, 12, 14) and boils down to the following operation: Train a simple regression model **δ** ≈ **y***β*_1_+*β*_0_ to remove the linear effect of age from the delta estimate. The new estimate

(8) $$\begin{array}{l} \delta_{1}=\delta -\text{y}\beta_{1}-\beta_{0} \end{array}$$

represents corrected brain age delta which is uncorrelated with age. We can inspect the value of *β*_1_ by taking the simple linear regression formula: $\beta_{1}=r_{\delta \text{y}}\frac{s_{\delta }}{s_{\text{y}}}$, where ***r***_δ*y*_ is age delta correlation (ADC) which is negative (Section 2.4). ***s***_δ_ is the standard deviation of the residuals and unnormalized square root of the residual sum of squares (RSS), *s*_**y**_ is the standard deviation of the responses and the unnormalized square root of the total sum of squares (TSS). In OLS, Ridge, and Kernel Ridge regression, we have TSS ≥ RSS. Hence, *β*_1_ ∈ [−1, 0]. Combining Equations (8) and (7) the entire model can be written in one equation as

(9) $$\begin{array}{l} \delta_{1}=\;f\left(\text{X}\right)-\text{y}\left(1+\beta_{1}\right)-\beta_{0} \end{array}$$

where (1+*β*_1_) ∈ [0, 1]. From Equation (9), we can see that the correction does not affect the predictions *f* (**X**). Instead, it implies shrinking **y**.

This approach is invalid in a predictive modeling framework because it *corrects the data* **y***, not the predictions* *f* (**X**). Beheshti et al. (12) report a lower error and a larger *R*^2^ value compared to approach 2 introduced in the next section. However, since this effect is obtained by shrinking the data it can be considered as an artifact of this approach.

#### 2.3.2. Approach 2: Scale up *f*(**X**) (Predicted age)

As an alternative approach, it has been suggested that $\hat{\text{y}}$ instead of ***δ*** should be used in the regression (1, 4). To this end, train a simple regression model $\hat{\text{y}}\approx \text{y}\beta_{1}+\beta_{0}$. Then define corrected predictions ${\hat{\text{y}}}_{2}$ as

(10) $$\begin{array}{l} {\hat{\text{y}}}_{2}=f\left(\text{X}\right)\beta_{1}^{-1}-\beta_{0}\beta_{1}^{-1} \end{array}$$

with corresponding brain age delta $\delta_{2}={\hat{\text{y}}}_{2}-\text{y}$. This modified age delta estimate is again uncorrelated with age. As in approach 1, the correction is performed using simple linear regression and we have $\beta_{1}=r_{ŷ\text{y}}\frac{s_{ŷ}}{s_{\text{y}}}$. *s*_ŷ_ is the standard deviation of the predictions and the unnormalized square root of the explained sum of squares (ESS). In OLS, Ridge, and Kernel Ridge regression, we have TSS ≥ ESS and *r*_ŷ*y*_ ∈ [0, 1]. This implies that *β*_1_ ∈ [0, 1]. Combining Equations (10) and (7) this can be written in one equation as

(11) $$\begin{array}{l} \delta_{2}=f\left(\text{X}\right)\beta_{1}^{-1}-\text{y}-\frac{\beta_{0}}{\beta_{1}} \end{array}$$

with $\beta_{1}^{-1}\;\in \;\left[1,\infty \right)$. Comparing Equations (9) and (11), we see that in approach 1 the data vector **y** is scaled down whereas in approach 2 the predictions *f* (**X**) are scaled up. In Section 2.6, we show that the two types of corrected residuals are actually identical up to scaling and therefore corr(***δ***_1_, ***δ***_2_) = 1. Consequently, they perform equally well on secondary analyses e.g., relating brain age delta to cognition. They are further closely related to our zero correlation constraint (Section 2.5.1). In a predictive modeling framework, we consider approach 2 as preferable since corrections should be applied to the model not to the data.

### 2.4. Negative Age Delta Correlation (ADC)

The theoretical basis for negative ADC has already been discussed in (14). In particular, the authors highlighted that ADC ≤ 0 for any sensible regression model. Here, we discuss ADC more specifically for the three regression models introduced above. We start with OLS regression. Let us expand the age delta correlation term as

(12) $$\begin{array}{l} \text{corr}\left(\text{y},\delta \right)=\frac{{\text{y}}^{⊤}\left(\hat{\text{y}}-\text{y}\right)}{\left\|\text{y}\|\;\|\hat{\text{y}}-\text{y}\right\|} \end{array}$$

where to simplify the notation we assume that **X** and **y** have been centered. The sign of the correlation is determined by the numerator. Defining **H** = **X**(**X**^⊤^**X**)^−1^**X**^⊤^ and writing $\hat{\text{y}}=\text{H}\text{y}$ we obtain

(13) $$\begin{array}{l} -{\text{y}}^{⊤}\text{y}+{\text{y}}^{⊤}\text{H}\text{y}=-{\text{y}}^{⊤}\left(\text{I}-\text{H}\right)\text{y}\leq 0 \end{array}$$

where the inequality follows from the fact that **I** − **H** is symmetric and idempotent and therefore positive semi-definite (29). This implies that the ADC is always non-positive in OLS regression. Ridge regression coincides with OLS regression for λ = 0. As λ increases, ***β*** tends to zero due to the shrinkage effect of the regularization (21). This implies that $\hat{\text{y}}\to 0$ and therefore corr(**y**, ***δ***) → −1 as λ increases. This is illustrated empirically in Figure 1C. The same argumentation holds for Kernel Ridge, one only has to replace **X** by Φ(**X**). Often, Kernel Ridge models will have a smaller prediction bias because their higher complexity allows for a better fit to the data. Furthermore, Figure 1D shows a clear bias toward large negative ADC values when regression coefficients are randomly sampled. Figure 1E shows that the bias persists in both train and test sets. Together, these results suggest that a negative ADC is inevitable and that regularization further exacerbates this effect, in line with previous work (3, 14).

### 2.5. Correlation Constraints for Regression

The regression problems defined in Section 2.2 can be cast as unconstrained optimization problems. The optimization involves the minimization of a *loss function* L which measures the amount of discrepancy between the true responses **y** and the model predictions $\hat{\text{y}}$:

(14) $$\begin{array}{l} \text{minimize }L\left(\text{y},\hat{\text{y}}\right). \end{array}$$

In OLS regression the loss function is the squared loss $L\left(\text{y},\hat{\text{y}}\right)=\|\text{y}-\hat{\text{y}}{\|}_{2}^{2}$ whereas it is $L\left(\text{y},\hat{\text{y}}\right)=\|\text{y}-\hat{\text{y}}{\|}_{2}^{2}+\lambda \|\beta_{1:p}{\|}_{2}^{2}$ in Ridge and Kernel Ridge regression. To control for age delta correlation in the training data, we can add a correlation constraint that caps the permitted magnitude of correlation between the brain age delta and age. To this end, consider the constrained optimization problem

(15) $$\begin{array}{l} \text{minimize }L\left(\text{y},\hat{\text{y}}\right)\\ \text{subject to }|\text{corr}\left(\text{y},\delta \right)|\leq \rho . \end{array}$$

The same loss function as before is minimized. However, the set of feasible solutions is limited to solutions for which the absolute value of the correlation does not exceed *ρ*, where *ρ* ≥ 0 is the correlation bound selected by the user. As a special case of Equation (15), we can consider the case *ρ* = 0, that is, the responses have to be perfectly uncorrelated with the residuals:

(16) $$\begin{array}{l} \text{minimize }L\left(\text{y},\hat{\text{y}}\right)\\ \text{subject to corr}\left(\text{y},\delta \right)=0. \end{array}$$

We will address the latter case first and see that it leads to a simple solution. In the following, we will assume that **X** and **y** have been centered and the model contains no intercept since this simplifies the equations. This does not limit the generality of our results. As shown in the Supplementary Material (Section 1), a model with centered data and without intercept yields the same regression coefficients as a model with intercept. In other words, we can first calculate ***β***_1:*p*_ on the centered data and subsequently calculate the intercept *β*_0_ to obtain the model for non-centered data.

#### 2.5.1. Zero Correlation Constraint

A hard correlation constraint can be set that requires the correlation between the residuals and the response values to be zero, that is |corr(**y**, *δ*)| = 0. In the Supplementary Material (Section 2) the optimal solution, ***b***, is derived for OLS, Ridge, and Kernel Ridge regression. It is given as a scaled version of the standard, unconstrained solution

(17) $$\begin{array}{l} {\text{b}}_{1:p}=\theta_{0}\beta_{1:p} \end{array}$$

where ***β*** is the standard OLS, Ridge, or Kernel Ridge solution and it is assumed that **X** and **y** have been centered. Using Equation (6), we can see that for Kernel Ridge regression this translates into a scaling of the dual weights

(18) $$\begin{array}{l} \alpha_{\rho }=\theta_{0}\alpha . \end{array}$$

The scaling factor *θ*_0_ is given by

(19) $$\begin{array}{l} \theta_{0}=\frac{\|\text{y}{\|}^{2}}{{\text{y}}^{⊤}\text{H}\text{y}} \end{array}$$

with model-specific hat matrices **H**:

$$\begin{array}{l} \text{H}=\;\text{X}{\left({\text{X}}^{⊤}\text{X}\right)}^{-1}{\text{X}}^{⊤}\;\text{(OLS)}\\ \text{H}=\;\text{X}{\left({\text{X}}^{⊤}\text{X}+\lambda \text{I}\right)}^{-1}{\text{X}}^{⊤}\;\text{(Ridge)}\\ \text{H}=\;\text{K}{\left(\text{K}+\lambda \text{I}\right)}^{-1}\;\text{(Kernel Ridge)}. \end{array}$$

*Intercept term*: If the data has not been centered and the model includes an intercept term, **y** and **y**^⊤^**Hy** need to be centered before calculating *θ*. The intercept ***b***_0_ can be obtained from the equation

$$\begin{array}{l} \left(\text{y}-1ȳ\right)=\;\left(\text{X}-1{\bar{\text{x}}}^{⊤}\right)\;{\text{b}}_{1:p}\\ \;\Leftrightarrow \;\text{y}=\;\text{X}{\text{b}}_{1:p}+1\left(ȳ-{\bar{\text{x}}}^{⊤}{\text{b}}_{1:p}\right) \end{array}$$

from which it follows that ${\text{b}}_{0}=ȳ-{\bar{\text{x}}}^{⊤}{\text{b}}_{1:p}$. The full correlation constrained model with intercept term is then given by the concatenation of the coefficients ***b*** = [***b***_0_, ***b***_1:*p*_].

#### 2.5.2. Bounded Correlation Constraint

The zero correlation solution successfully removes the correlation between residuals and responses. However, it does so at the cost of goodness of fit to the training data. Furthermore, in predictive modeling, perfect control of ADC on the training set is less important than good predictive performance and low bias on the test set. This suggests the need for a softer constrained optimization solution wherein the equality constraint is replaced by an inequality constraint. In the Supplementary Material (Section 3) it is shown that the optimal solution is again given by scaling, ***b***_1:*p*_ = *θ_ρ_**β***_1:*p*_, where there are now two possible solutions for the scaling factor,

$$\begin{array}{l} \theta_{1,2}=\|\text{y}{\|}^{2}\;{\text{y}}^{⊤}\hat{\text{y}}\left(1-\rho^{2}\right)/c\\ \;\pm \frac{\|\text{y}{\|}^{2}}{\left|c\right|}\sqrt{\rho^{2}\left(1-\rho^{2}\right)\left(\|\text{y}{\|}^{2}\|\hat{\text{y}}{\|}^{2}-{\left({\text{y}}^{⊤}\hat{\text{y}}\right)}^{2}\right)} \end{array}$$

where $c={\left({\text{y}}^{⊤}\hat{\text{y}}\right)}^{2}-\rho^{2}\|\text{y}{\|}^{2}\|\hat{\text{y}}{\|}^{2}$ and $\hat{\text{y}}$ is the predictions under the unconstrained OLS, Ridge, or Kernel Ridge model. The two solutions define an interval [*θ*_1_, *θ*_2_]. Setting *θ* to any value within this interval will guarantee −*ρ* ≤ corr(**y**, *δ*_cc_) ≤ ρ, where *δ*_cc_ is the brain age delta under the correlation constrained models. Setting θ = θ_1_ or θ = θ_2_ will set corr(**y**, δ_cc_) = −*ρ* or corr(**y**, *δ*_cc_) = *ρ*, respectively. From this we can derive the following algorithm for correlation constrained models with an inequality constraint:

1. Calculate the standard, unconstrained solution for the model (OLS, Ridge, or Kernel Ridge). If |corr(**y**, *δ*)| ≤ *ρ*, the unconstrained solution does not violate the correlation constraints so we are done.
2. If |corr(**y**, *δ*)| > *ρ*, calculate θ_1, 2_ and set θ to the value that smaller in absolute value. This will assure that corr(**y**, δ_cc_) = −*ρ* if corr(**y**, δ) < −*ρ*.

**Figure 2:**
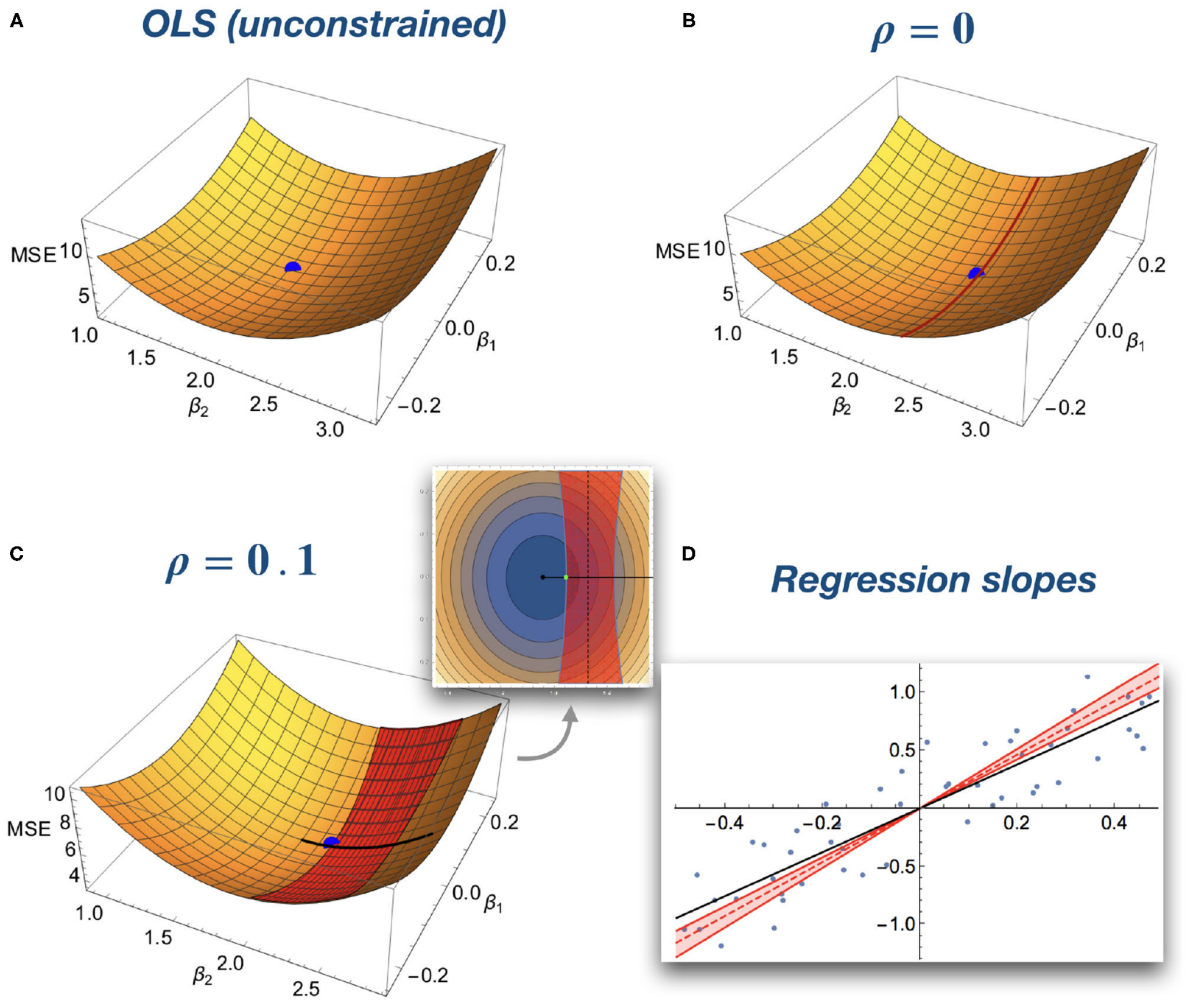
Geometrical intuition of optimization of an OLS model with correlation constraints. For illustrative purposes, simulated data is used with a model with two regression coefficients and without intercept. **(A)** x and y axes represents different combinations of regression coefficients, whereas the z-axis represents the residual sum of squares. The standard unconstrained OLS fit corresponds to the minimum of the quadratic surface (blue ball). **(B)** Zero correlation constraint. The solution space is restricted to a hyperplane (red line on the surface). The blue dot represents the optimal constrained solution. **(C)** Bounded correlation. The solution space is restricted to the space between two paraboloids (red band on the surface). The inset depicts a “top down” view on the surface. **(D)** Corresponding regression slopes. The OLS solution is depicted as a black line. The correlation bound allows for a set of solutions (red shaded area) limited by *ρ* = −0.1 and *ρ* = 0.1 (red solid lines). The zero ADC solution is depicted as a red dashed line. All constrained solutions have a larger slope than the OLS solution.

Figure 2 depicts the geometrical intuition underlying the correlation constraints. Without constraints, the solution is the minimum of a quadratic function (Figure 2A). For a zero correlation constraint, the set of feasible solutions is reduced to a line within this space (Figure 2B). For a bounded correlation constraint, the set of feasible solutions is the space between two paraboloids (Figure 2C). In both cases, the correlation constraints lead to a larger slope for the regression coefficients as compared to the unconstrained model (Figure 2D).

### 2.6. Relationship Between Zero Correlation Constraint and Existing Correction Approaches

In Section 2.3, we reviewed the two main approaches for correcting brain age delta used in the literature. Here, we investigate their mutual relationship as well as their relationship to our approach. Without loss of generality we assume that **y** and **X** have been centered (see Supplementary Material, Section 1). Let us start from Equation (8) corresponding to approach 1. The regression slope β_1_ for a simple linear regression model is given by

$$\beta_{1}=\text{corr}\left(\delta ,\text{y}\right)\frac{\left\|\delta \right\|}{\left\|\text{y}\right\|}.$$

Writing $\delta =\hat{\text{y}}-\text{y}$ and expanding the correlation term as in Equation (S5) (Supplementary Material), we find that

$$\beta_{1}=\frac{{\text{y}}^{⊤}\text{H}\text{y}}{\|\text{y}{\|}^{2}}-1={\theta_{0}}^{-1}-1$$

with *θ*_0_ as defined in Equation (19). Therefore, the solution to Equation (8) is given by

(20) $$\begin{array}{l} \delta_{1}=\hat{\text{y}}-{\theta_{0}}^{-1}\text{y}. \end{array}$$

Alternatively, using approach 2 (correction of predictions) and starting from Equation (10) we perform a regression of $\hat{\text{y}}$ on **y**. Again, this is a simple linear regression model whose slope is given by

$$\beta_{1}=\text{corr}\left(\hat{\text{y}},\text{y}\right)\frac{\left\|\hat{\text{y}}\right\|}{\left\|\text{y}\right\|}={\theta_{0}}^{-1}$$

yielding the corrected predictions ${\hat{\text{y}}}_{2}=\hat{\text{y}}/\beta_{1}=\theta_{0}\hat{\text{y}}$ with corresponding brain age delta

(21) $$\begin{array}{l} \delta_{2}=\theta_{0}\;\hat{\text{y}}-\text{y}. \end{array}$$

This solution uses a scaling of $\hat{\text{y}}$ by θ_0_ and is thus equivalent to our zero correlation solution when an OLS model is used. Furthermore, comparing Equations (20) and (21) we see that both solutions are proportional to each other. Their relationship is given by

(22) $$\begin{array}{l} \delta_{2}=\theta_{0}\;\delta_{1} \end{array}$$

and therefore corr(*δ*_1_, *δ*_2_) = 1. In other words, the brain age delta estimates from the two approaches used in the literature are identical up to a scaling factor of *θ*_0_.

### 2.7. Interpretability

Maximizing predictive performance is the primary objective when optimizing statistical models. To empirical researchers, understanding *what* the regression model learns from the data is useful, too. Linear regression models such as OLS and Ridge can be interpreted in terms of their ***β*** coefficients. To keep the notation simple, we will assume that the data has been demeaned and the model contains no intercept term. If the data is standardized, large components of the ***β*** can be interpreted as features that are relevant to the regression task. Since our models combine prediction and correction into a single task, the ***β***’s capture the entire operation of the model. Importantly, the choice of the correlation bound ρ does not change the interpretation. Since the regression coefficients in our models are just scaled versions of the original regression coefficients, ***b*** = *θ**β*** for some *θ* ∈ ℝ, the choice of *ρ* does not affect the ratio between any pair of coefficients.

For collinear data, coefficients can become uninterpretable with large weights for features that are not related to the target variable. In this case, *structure coefficients* (18) and *activation patterns* (19) have been proposed as alternatives metrics. An activation pattern is given by

$${\text{a}}_{\rho }=\Sigma \;\text{b}$$

where ${\text{a}}_{\rho }\;\in {\mathbb{R}}^{p}$ is the activation pattern and **Σ** is the data covariance matrix. Let ***a***_***β***_ = **Σ** ***β*** be the activation pattern for the standard (uncorrected) model. Then setting ρ merely scales the activation pattern by *θ_ρ_* since ***a***_*ρ*_ = **Σ *b*** = **Σ** *θ_ρ_* ***β*** = *θ*_*ρ*_ ***a***_***β***_. An example for an activation pattern is depicted in **Figure 5** and discussed in Section 3.3.

Structure coefficients are given by the vector of Pearson correlations between $\hat{\text{y}}=\text{X}\text{b}$ and each column of **X**, where the *i*-th column is denoted as **X**_*i*_. Since the correlation coefficient is invariant to constant shifts and scaling, we have corr(**X**_*i*_, **X*b***) = corr(**X**_*i*_, **X*****β***), that is, structure coefficients are invariant to the choice of *ρ*.

For kernel methods, these coefficients are generally not available. As an alternative, we considered the vector of *partial derivatives* of regression model with the respect to each of the features,

$$\partial_{\text{i}}f\left(\text{x}\right)={\left(\partial_{\text{i}}k\left(\text{x}\right)\right)}^{⊤}\alpha ,$$

where *k* is a kernel function (20) and *i* refers to the index of the feature. Using Equation (18), we see that for a correlation constrained Kernel Ridge model *f*_ρ_ these partial derivatives are again just scaled versions of the unconstrained version ∂_*i*_*f*_*ρ*_(**x**) = *θ_ρ_*∂_*i*_*f* (**x**).

To summarize, our models are interpretable because the regression coefficients capture the whole operation of the model. Furthermore, the choice of the correlation bound does not affect the interpretation of the model: structure coefficients are invariant to the choice of *ρ*, whereas regression coefficients, activation patterns and partial derivatives are merely scaled by a constant factor.

### 2.8. Toolbox

The Linear, Ridge, and Kernel Ridge regression models with correlation constraints presented in this paper have been implemented in Python and MATLAB. For Python, the models are available on GitHub (github.com/treder/correlation-constrained-regression). They inherit from and are fully compatible with the Scikit-Learn framework (30). The models extend Scikit-Learn's LinearRegression, Ridge, and KernelRidge models with an additional parameter correlation_bound that corresponds to *ρ* in Equations (15) and (16). Setting the parameter to 0 enforces a zero correlation constraint whereas setting it to a positive value bounds the correlation accordingly. For MATLAB, the models have been integrated into MVPA-Light (31), an open-source machine learning toolbox. By setting the hyperparameter correlation_bound, ADC can be controlled in the same way as for the Python-based models. Code examples for both Python and MATLAB can be found on the GitHub page.

### 2.9. Neuroimaging Data

Neuroimaging data supplied within the Predictive Analytics Competition were fully pre-processed T1-weighted MRI scans from 2,640 training set and 660 validation set subjects as described previously (2). All normalized 3D maps of gray matter (GM) and white matter (WM) volume were used to create group GM and WM masks. Each GM and WM image was smoothed using an 8-mm Gaussian kernel, masked and concatenated into a vector of 153,237 and 86,143 voxels, respectively.

Concatenated GM and WM images were intensity normalized and submitted to Independent Component Analysis using the Group ICA of fMRI toolbox [https://trendscenter.org/software/gift/; (32)]. The optimal number of components of the ICA decomposition (72 and 99 for GM and WM images, respectively) was determined using Principal Component Analysis (PCA) with minimum description length (MDL) model order selection criteria (33). Normalized features in the training model, based on 2,640 participants, included scores for all GM and WM components (*N* = 171). Additional covariates included total GM, total WM, gender and dummy coding for 17 scanning sites. We also included low-order interaction terms (such as bivariate interaction) between total GM, total WM, gender, PC1 and PC2 scores.

### 2.10. Brain Age Prediction

We performed brain age prediction using Python with Scikit-Learn and our custom extensions. The models were tested in three different conditions: *Unconstrained* (using Scikit-Learn's models without correlation constraints), *zero correlation* (*ρ* = 0) (using our extensions with a correlation constraint of 0), *bounded correlation* (*ρ* = 0.1, 0.2, 0.3) (using our extensions with a correlation bound of 0.1, 0.2, 0.3), and approaches 1 and 2 from the literature introduced in Section 2.3. To obtain both in-sample and out-of-sample statistics, models were applied to both training and test data. To estimate the variability of predictive performance, we performed 100 iterations. In every iteration, training data was randomly sampled from the training set using bootstrapping. The 171 Independent Components were used as features.

Three regression models were considered, OLS, Ridge regression, and Kernel Ridge regression with a RBF kernel. For both Ridge and Kernel Ridge regression, hyperparameters were tuned using a grid search with Scikit-Learn's GridSearchCV and five-fold cross-validation. For Ridge regression, the regularization parameter was tuned using candidate values α = (10^−3^, 10^−2^, 10^−1^, 1, 10). For Kernel Ridge with a RBF kernel, kernel width γ = (100, 10, 1, 10^−1^) and α = (10^−3^, 10^−2^, 10^−1^, 1, 10) were tuned. The resultant best model was used to calculate in- and out-of-sample metrics. Mean absolute error (MAE) and age delta correlation (ADC) served as metrics. Denoting train and test sets as TR and TE, MAE was estimated as

(23) $$\begin{array}{l} {\text{MAE}}_{\text{train}}=\frac{1}{\left|\mathrm{TR}\right|}\underset{i\in \mathrm{TR}}{\sum }|{\text{y}}_{i}-f\left({\text{x}}_{i}\right)|\\ \;{\text{MAE}}_{\text{test}}=\frac{1}{\left|\mathrm{TE}\right|}\underset{i\in \mathrm{TE}}{\sum }|{\text{y}}_{i}-f\left({\text{x}}_{i}\right)| \end{array}$$

where *f* is a regression model trained on the training data and |TR| and |TE| are the sizes of the train and test sets, respectively. Similarly, ADC was calculated separately for the predictions in the train and test sets. All analyses were performed on a Desktop computer with an Intel Core i7-6700 @ 3.40 GHz x 8 CPU with 64 GB RAM running on Ubuntu 18.04. The analysis code is available as part of the toolbox^2^.

## 3. Results

### 3.1. Brain Age Prediction

**Figure 3:**
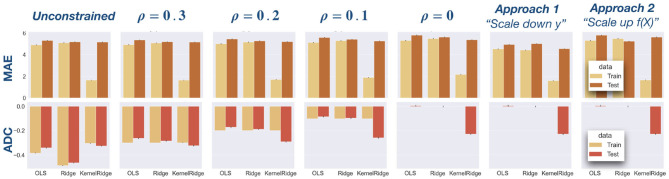
Analysis of PAC data using OLS, Ridge, and Kernel Ridge regression models. In Table 2, this data is show in tabular form. **First row:** Mean absolute error (MAE) is depicted for the three regression models and different constraints: unconstrained (standard regression model), bounded correlation (*ρ* = 0.1, 0.2, 0.3), zero correlation (*ρ* = 0), and approaches 1 and 2 from the literature (see Section 2.3). In terms of MAE, the best model on both train and test sets is Kernel Ridge regression. **Second row:** Age delta correlation (ADC) for different models and correlation constraints. Our models exactly control ADC on the training data and also reduce ADC on test data.

Figure 3 depicts the MAE and ADC results on the PAC data for train and test sets separately, comparing the three regression models (OLS, Ridge, and Kernel Ridge regression) and different constraints on the age delta correlation (ADC): unconstrained (standard regression model), bounded correlation constraint (*ρ* = 0.1, 0.2, 0.3), zero correlation constraint (*ρ* = 0), and approaches 1 and 2 from the literature introduced in Section 2.3. The same data is presented in tabular form in Table 2. Bonferroni correction was used in case of multiple comparisons.

**Table 2:** Mean absolute error (MAE) and age delta correlation (ADC) for different types of correlation constraints and regression models.

**Data**	**Constraint**	**OLS**	**Ridge**	**Kernel ridge**
MAE (Train)	Unconstrained	4.91 ± 0.09	5.1 ± 0.08	1.65 ± 0.04
	*ρ* = 0.3	4.93 ± 0.09	5.09 ± 0.08	1.65 ± 0.03
	*ρ* = 0.2	5.0 ± 0.09	5.16 ± 0.1	1.7 ± 0.04
	*ρ* = 0.1	5.12 ± 0.1	5.29 ± 0.1	1.89 ± 0.05
	*ρ* = 0	5.3 ± 0.11	5.48 ± 0.11	2.17 ± 0.06
	Approach 1	4.52 ± 0.08	4.43 ± 0.07	1.59 ± 0.03
	Approach 2	5.3 ± 0.1	5.48 ± 0.11	1.67 ± 0.04
MAE (Test)	Unconstrained	5.31 ± 0.09	5.18 ± 0.06	5.16 ± 0.09
	*ρ* = 0.3	5.35 ± 0.09	5.19 ± 0.07	5.16 ± 0.09
	*ρ* = 0.2	5.44 ± 0.1	5.27 ± 0.07	5.19 ± 0.09
	*ρ* = 0.1	5.59 ± 0.1	5.42 ± 0.08	5.26 ± 0.1
	*ρ* = 0	5.79 ± 0.11	5.62 ± 0.09	5.37 ± 0.11
	Approach 1	4.95 ± 0.07	4.99 ± 0.09	4.54 ± 0.06
	Approach 2	5.82 ± 0.09	5.22 ± 0.1	5.64 ± 0.08
ADC (Train)	Unconstrained	−0.384 ± 0.007	−0.486 ± 0.007	−0.305 ± 0.006
	*ρ* = 0.3	−0.3 ± 0.0	−0.3 ± 0.0	−0.299 ± 0.002
	*ρ* = 0.2	−0.2 ± 0.0	−0.2 ± 0.0	−0.2 ± 0.0
	*ρ* = 0.1	−0.1 ± 0.0	−0.1 ± 0.0	−0.1 ± 0.0
	*ρ* = 0	0.0 ± 0.0	0.0 ± 0.0	0.0 ± 0.0
	Approach 1	0.0 ± 0.0	0.0 ± 0.0	0.0 ± 0.0
	Approach 2	0.0 ± 0.0	0.0 ± 0.0	0.0 ± 0.0
ADC (Test)	Unconstrained	−0.341 ± 0.021	−0.464 ± 0.018	−0.325 ± 0.022
	*ρ* = 0.3	−0.263 ± 0.021	−0.284 ± 0.021	−0.324 ± 0.023
	*ρ* = 0.2	−0.173 ± 0.022	−0.19 ± 0.022	−0.291 ± 0.023
	*ρ* = 0.1	−0.083 ± 0.023	−0.095 ± 0.022	−0.26 ± 0.024
	*ρ* = 0	0.005 ± 0.023	−0.001 ± 0.022	−0.228 ± 0.024
	Approach 1	0.005 ± 0.023	−0.001 ± 0.022	−0.228 ± 0.024
	Approach 2	0.005 ± 0.023	−0.001 ± 0.022	−0.228 ± 0.024

*In Figure 3, this data is depicted as bar graphs*.

With respect to mean absolute error (MAE), a significantly lower error was found on the train data compared to test data (Wilcoxon signed-rank test, *W* = 62, 449, *p* < 0.0001). For the constrained models, we performed a regression analysis of MAE on *ρ*. We found significant negative slopes for all models and train and test sets separately (all *p* < 0.0001), indicating that MAE decreases significantly as *ρ* increases. On the test data, we used Wilcoxon signed-rank tests to compare MAE for the unconstrained model with MAE for each of the constrained models. A significantly higher MAE was obtained for most unconstrained models (all *p* < 0.0001). The only exception was approach 1 (“scale down **y**”) wherein the relationship is reversed: MAE decreases after correction. This is an artifact of the fact that the correction is applied to the data, not the predictions (see Section 2.3).

With respect to age delta correlation (ADC), our models perfectly controlled for ADC on the training data. ADC was significantly larger in magnitude for the test data compared to the train data (*W* = 877, 894, *p* < 0.0001). Linear regression of ADC on *ρ* showed a significant negative slope for all models on both train and test sets (all *p* < 0.001). On the test set, ADC was significantly larger in magnitude for the unconstrained model than for the constrained models (all *p* < 0.0001), suggesting that the constrained models also control ADC on the test set.

### 3.2. ADC-MAE Trade-Off

**Figure 4:**
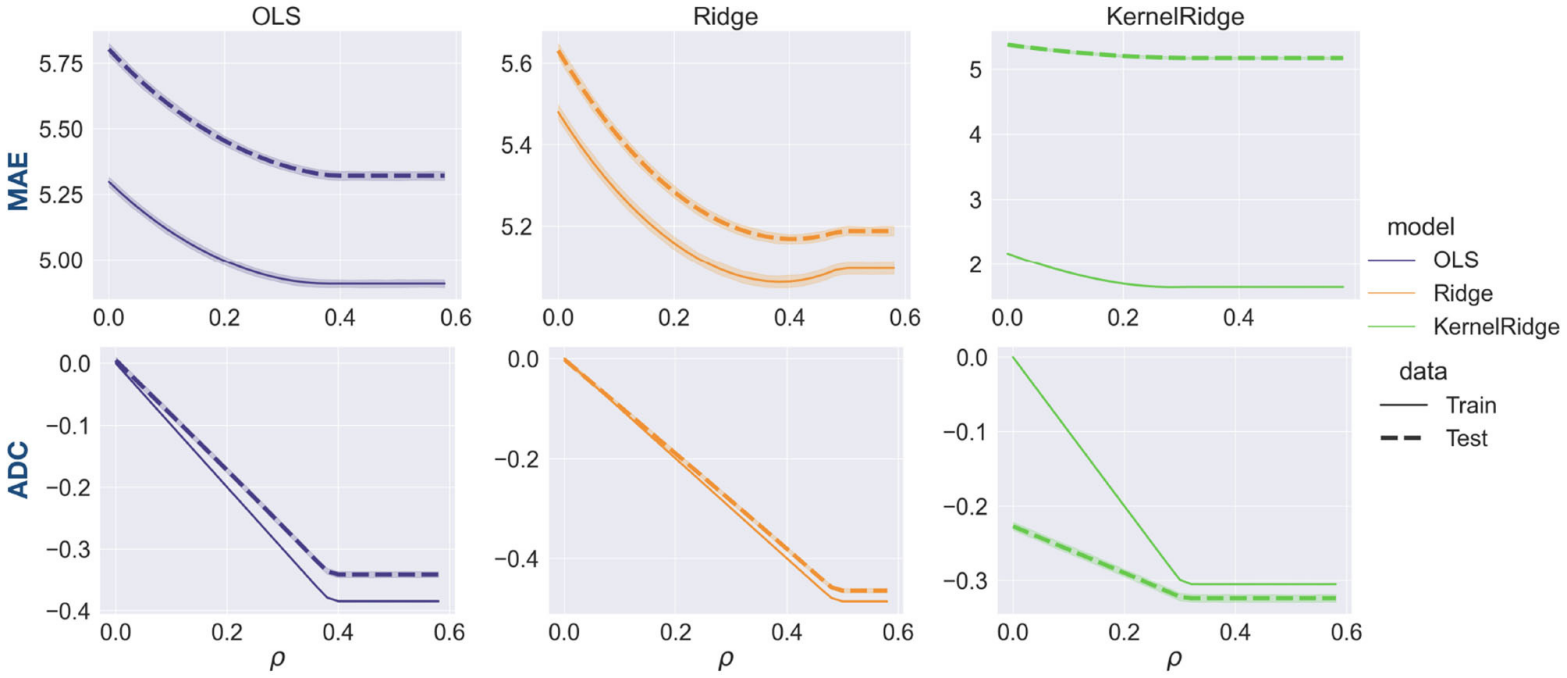
Trade-off between MAE (row 1) and ADC (row 2) as a function of the hyperparameter *ρ* that represents the correlation bound in our models. The shaded area around the lines represents standard deviation. Increasing *ρ* leads to a lower MAE but this comes at the expense of ADC increasing in magnitude. MAE and ADC change in a similar way on training and test sets, suggesting that the training set is a good proxy for test set performance.

To better characterize how the choice of *ρ* mediates the trade-off between ADC and MAE, we repeated the prediction analysis. This time the correlation bound was varied in small steps of 0.02. Results averaged across 100 bootstrap iterations are depicted in Figure 4. In line with the results above, MAE generally decreases with increasing *ρ* and eventually flattens off. ADC decreases roughly linearly with ρ. It flattens off at a value of *ρ* that corresponds to the ADC value of the uncorrected model, since no correction needs to be applied when *ρ* > |ADC|. Furthermore, MAE and ADC change similarly for train and test set, albeit with different slopes. This is a useful observation since it suggests that the hyperparameter *ρ* can be optimized on the training set alone, in line with good practice in predictive modeling.

### 3.3. Interpretability via Activation Patterns

**Figure 5:**
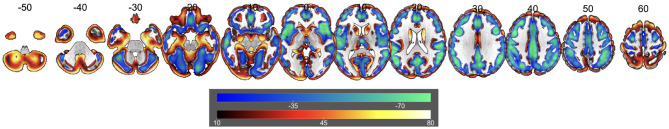
Activation pattern for an OLS model trained on the gray matter Independent Components (ICs). Warms colors correspond to positive values and cold colors to negative values. The brain map has been produced by multiplying each entry of the activation pattern with the map corresponding to each IC, and then summing up all the maps. The choice of *ρ* does not affect the map in relative terms, it only affects its scaling.

Figure 5 shows an activation pattern for an OLS model with *ρ* = 0 trained on the whole training set, using gray matter Independent Components (ICs) only. To create the maps, a vector of activation patterns ***a*** was calculated for the gray matter ICs (see Section 3.3). Since each IC corresponds to a brain map, we multiplied each entry of ***a*** with its corresponding map and added up the maps. The figure depicts the resultant summed map indicating an age-related decrease in intensity values in deep cortical areas. An age-related increase in intensity values was observed at the boundaries between gray matter and other tissue types, likely reflecting CSF signals in older adults (34). Crucially, the map is independent of the choice of ρ, only its scaling is affected by ρ. This illustrates that the interpretation of the models is not affected by a change of ρ.

## 4. Discussion

A predictive bias manifesting as an overprediction of the age of young individuals and an underprediction of the age of elderly individuals has been consistently reported in the brain age literature (2, 3, 14, 15). It can be quantified as age delta correlation (ADC), that is, the correlation between brain age delta (predicted age minus chronological age) and chronological age. We introduced modifications to three popular regression models, OLS, Ridge and Kernel Ridge regression, that effectively control ADC. To this end, we introduced a hyperparameter *ρ* that can be set by the user. It represents a correlation bound that controls the maximum permissible ADC. The resultant models are optimal in that they give the lowest mean-squared error on the training set (among all models from the same class) while controlling for ADC.

Our models were tested on the PAC competition data using different values for *ρ*. The models not only perfectly controlled ADC on the training data, they also approximately controlled ADC on unseen test data (see Figure 3). For all constrained models, ADC on the test set was lower than for the unconstrained models. The flip side of this was an increase of mean absolute error (MAE) for our constrained models as compared to the unconstrained model, but often this increase was modest. For instance, for an OLS model MAE increased from 5.31 for the unconstrained model to 5.35 for the model with *ρ* = 0.3, an increase of only 0.8%. Across all models in the test set, we found that an increase in *ρ* led to a decrease in MAE. This suggests that *ρ* can be used as a lever to finely control the trade-off between predictive performance (MAE) and age delta correlation (ADC).

In the same analysis, we included the two existing correction methods used in the literature, denoted as approach 1 (3, 12, 14) and approach 2 (1, 4) discussed in detail in Section 2.3. For the special case of using a OLS model with a zero correlation constraint, *ρ* = 0, our models' brain age deltas are equivalent to approach 2. In Section 2.6, we furthermore show that the brain age deltas in approaches 1 and 2 are actually identical up to scaling. They differ only by the scaling factor θ_0_ defined in Equation (19). In particular, in approach 1 chronological age is scaled down by this factor before calculating brain age delta, whereas in approach 2 *f* (**X**) (predicted age) is scaled up by the same amount. The downscaling in approach 1 leads to a lower MAE which is even smaller than for an uncorrected model. We would like to stress that this is because approach 1 corrects the data, not the predictions. This is not permissible in predictive modeling and approach 2 should be preferred.

The hyperparameter *ρ* controlling ADC has to be selected by the user. Figure 4 shows how the choice of *ρ* affects both MAE and ADC. A possible selection criterion would involve defining a maximum permissible MAE or ADC value and choosing *ρ* accordingly. A more comprehensive analysis would take into account any follow-up analyses. For instance, brain age delta is often correlated with cognitive variables in a second step. An optimal selection of *ρ* could involve e.g., the regression slope or *p*-value of the association on the training set. A detailed exploration of how the choice of *ρ* affects follow-up analyses requires a dataset including cognitive test scores and is left for future work.

In terms of interpretability, our models offer a greater degree of transparency than the traditional two approaches because the model coefficients capture the entire brain age prediction pipeline (i.e., both prediction and correction). Moreover, in Section 2.7 we show that the interpretation is not affected by changes in ρ, a hyperparameter in our models. Structure coefficients (18) are invariant to changes in ρ, whereas the other metrics are simply scaled by a constant value.

**Figure 6:**
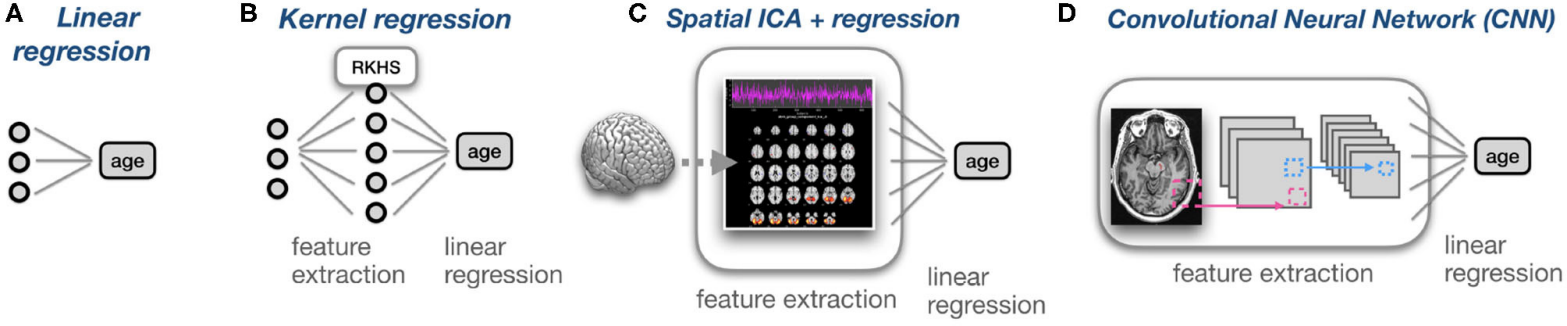
Coupled with non-linear feature extraction techniques, linear regression becomes a powerful model that is implicitly a part of many regression models. **(A)** Linear regression using three features to predict age. **(B)** Kernel regression can be conceived of as a projection of the data into a higher-dimensional, Reproducing Kernel Hilbert Space (RKHS), followed by linear regression in this space. **(C)** MRIs can be transformed into components using ICA. For each MRI, its loadings on the different Independent Components can be used as features for linear regression. **(D)** In Convolutional Neural Networks (CNN) for regression, the last layer is typically linear. The preceding layers can be conceived of as feature extraction layers, and the last layer performs linear regression on non-linear features.

A limitation of our study is that it only covers OLS, Ridge, and Kernel Ridge regression. In the age of deep neural networks, the focus on linear and kernel methods may seem very limiting. Therefore, we would like to emphasize that linear regression models lie at the heart of many non-linear approaches including Convolutional Neural Networks. As illustrated in Figure 6, non-linear approaches can often be conceived of as linear regression operating on non-linearly extracted features. Linear models can uniquely combine predictive power with computational efficiency and interpretability. In line with this, (35) found that kernel regression was as performant as deep neural networks when predicting phenotypes from functional connectivity data. Nevertheless, future work could address the incorporation of correlation constraints into other models classes such as Lasso (36), Support Vector Regression (37) or CNNs. Since these models use iterative optimization, a possible approach could be adding the correlation term −corr^2^(**y**, *δ*) directly to the loss function. Alternatively, since **y** is constant this can be simplified to the quantity ${\text{y}}^{⊤}\hat{\text{y}}/\|\delta {\|}^{2}$.

On a more speculative note, future development of the brain age delta metric might benefit from work on errors-in-variables models (38–40) or measurement error models (16). Standard linear regression models assume that chronological age has been measured with an error whereas the brain data is noisefree. It is more likely that the opposite is true: chronological age can be measured with high accuracy but there is noise and individual variability in the brain scans. Not accounting for measurement error in the features leads to regression dilution which in OLS regression manifests as an underestimation of the regression coefficients. This phenomenon is known in the brain age literature (3, 14). Our scaling factor θ inflates the regression coefficients and therefore un-dilutes the model, but it is not clear to the authors whether there is a more formal relationship between correction of the residuals and measurement error. Unfortunately, estimating measurement error in brain scans requires repeated sampling which is often not available.

Concluding, without accurate control for ADC, the use of brain age delta can lead to false associations with other phenotypes and limit our understanding of the processes that underpin brain aging. We highlighted the importance of estimating brain age delta and controlling for age delta correlation within a given model, as we introduced a novel class of regression models that allow for fine control of ADC. Our solution is optimal on the training set and shows approximate control of ADC on the test set. In an era of “big data” predictive modeling, this approach nicely dovetails with strategies to develop reliable models that generalize to independent test sets for use in personalized and precision medicine (41).

## Data Availability Statement

The neuroimaging dataset analyzed in this study has been provided by the PAC 2019 team. All the program code and functions can be found in the following repository: https://github.com/treder/correlation-constrained-regression.

## Author Contributions

MT conceptualized the approach and developed the toolboxes for Python and MATLAB. KT processed the MRI data. MT and KT performed the age analyses. MT and JS performed the mathematical analysis. MT and KT wrote the manuscript with additional contributions, revision notes and comments from all authors.

## Conflict of Interest

The authors declare that the research was conducted in the absence of any commercial or financial relationships that could be construed as a potential conflict of interest.

## Footnotes

^1^www.frontiersin.org/research-topics/13501/predicting-chronological-age-from-structural-neuroimaging-the-predictive-analytics-competition-2019

^2^https://github.com/treder/correlation-constrained-regression/blob/main/run_regression_analysis.py
