# Supplementary material for: Correlation Constraints for Regression Models: Controlling Bias in Brain Age Prediction
Source: Front Psychiatry. 2021 Feb 18;12:615754. doi: 10.3389/fpsyt.2021.615754 (PMC7930839; doi:10.3389/fpsyt.2021.615754)
Supplement: Supplementary file 1 [file Presentation_1.pdf]

Supplementary Material

1 EQUAL COEFFICIENTS FOR MODELS WITH AND WITHOUT INTERCEPT

The coefficients vector $\boldsymbol{\beta}_{1:p}$ (excluding the intercept $\boldsymbol{\beta}_0$) obtained from a model including an intercept is the same as the $\boldsymbol{\beta}$ obtained from a model without an intercept but where the $\mathbf{X}$ and $\mathbf{y}$ have been centered. To prove this, let us start with Ridge regression. First, let us consider a model without intercept where we are centering the data using $\bar{\mathbf{x}} = \frac{1}{n}\mathbf{X}^\top \mathbf{1} \in \mathbb{R}^p$ (column means of $\mathbf{X}$) and $\bar{y} = \frac{1}{n}\mathbf{y}^\top \mathbf{1} \in \mathbb{R}$ (mean of $\mathbf{y}$). The loss function and its gradient are given by

$$\mathcal{L}(\boldsymbol{\beta}) = ||(\mathbf{y} - \mathbf{1}\bar{y}) - (\mathbf{X} - \bar{\mathbf{x}}\mathbf{1}^\top)\boldsymbol{\beta}\,||_2^2 + \lambda\,||\boldsymbol{\beta}||_2^2$$
$$\nabla_{\boldsymbol{\beta}}\,\mathcal{L}(\boldsymbol{\beta}) = 2(\mathbf{X} - \bar{\mathbf{x}}\mathbf{1}^\top)^\top[(\mathbf{X} - \bar{\mathbf{x}}\mathbf{1}^\top)\boldsymbol{\beta} - (y - \mathbf{1}\bar{y})] + 2\lambda\boldsymbol{\beta}.$$

Setting the gradient to zero and rearranging we arrive at

$$(\mathbf{X}^\top\mathbf{X} - n \cdot \bar{\mathbf{x}}\bar{\mathbf{x}}^\top + \lambda\mathbf{I})\,\boldsymbol{\beta} = (\mathbf{X} - \bar{\mathbf{x}}\mathbf{1}^\top)\,(\mathbf{y} - \bar{y}\mathbf{1}). \tag{S1}$$

Conversely, let us assume that the data has not been centered and the model includes an intercept $\boldsymbol{\beta}_0$, that is $\boldsymbol{\beta} = [\boldsymbol{\beta}_0, \boldsymbol{\beta}_{1:p}]$. We will not augment $\mathbf{X}$ with a column of ones but instead write the intercept explicitly. This leads to the loss function

$$\mathcal{L}(\boldsymbol{\beta}) = ||\mathbf{y} - \mathbf{X}\boldsymbol{\beta}_{1:p} - \mathbf{1}\,\boldsymbol{\beta}_0\,||_2^2 + \lambda\,||\boldsymbol{\beta}_{1:p}||_2^2.$$

The gradients w.r.t to $\boldsymbol{\beta}_0$ and $\boldsymbol{\beta}_{1:p}$ are given by

$$\nabla_{\boldsymbol{\beta}_0}\,\mathcal{L}(\boldsymbol{\beta}) = 2 \cdot \mathbf{1}^\top(\mathbf{X}\boldsymbol{\beta}_{1:p} + \mathbf{1}\,\boldsymbol{\beta}_0 - \mathbf{y})$$
$$= 2 \cdot n\,(\bar{\mathbf{x}}^\top\boldsymbol{\beta}_{1:p} + \boldsymbol{\beta}_0 - \bar{y})$$
$$\nabla_{\boldsymbol{\beta}_{1:p}}\,\mathcal{L}(\boldsymbol{\beta}) = 2\mathbf{X}^\top(\mathbf{X}\boldsymbol{\beta}_{1:p} + \mathbf{1}\,\boldsymbol{\beta}_0 - \mathbf{y}) + 2\lambda\boldsymbol{\beta}_{1:p}.$$

Setting both gradients to zero yields the solution for $\boldsymbol{\beta}_0$

$$\boldsymbol{\beta}_0 = \bar{y} - \bar{\mathbf{x}}^\top\boldsymbol{\beta}_{1:p}$$

which can be inserted into the gradient for $\boldsymbol{\beta}_{1:p}$

$$\begin{aligned}(\mathbf{X}^\top\mathbf{X} + \lambda\mathbf{I})\,\boldsymbol{\beta}_{1:p} + \bar{\mathbf{x}}\,\boldsymbol{\beta}_0 &= \mathbf{X}^\top\mathbf{y} \\ \iff (\mathbf{X}^\top\mathbf{X} - n \cdot \bar{\mathbf{x}}\bar{\mathbf{x}}^\top + \lambda\mathbf{I})\,\boldsymbol{\beta}_{1:p} &= \mathbf{X}^\top\mathbf{y} - n \cdot \bar{\mathbf{x}}\bar{y}.\end{aligned} \tag{S2}$$

Comparing Eq. (S1) and Eq. (S2) we can see that the solutions for models with and without intercept are identical. This result immediately generalizes to OLS (set $\lambda = 0$) and Kernel Ridge regression (replace $\mathbf{X}$ by $\Phi(\mathbf{X})$).

2 ZERO CORRELATION CONSTRAINT

Here, we use the Lagrangian multipliers approach to derive the solution for a zero correlation constraint. We will start with the OLS model and then show the equivalent results in Ridge and Kernel Ridge regression. In matrix notation, the constrained OLS optimization problem can be written as

$$\begin{aligned} \text{minimize} \quad & \frac{1}{2}||\mathbf{y} - \mathbf{X}\boldsymbol{\beta}||^2 \\ \text{subject to} \quad & \text{corr}(\mathbf{y}, \mathbf{y} - \hat{\mathbf{y}}) = 0 \end{aligned}$$

where the scaling factor $\frac{1}{2}$ does not affect the solution. Note that $\text{corr}(\mathbf{y}, \mathbf{y} - \hat{\mathbf{y}}) = 0$ implies zero covariance, that is $\text{cov}(\mathbf{y}, \mathbf{y} - \hat{\mathbf{y}}) = 0$. If we expand the covariance term the constraint simplifies to

$$||\mathbf{y}||^2 - \mathbf{y}^\top \mathbf{X}\boldsymbol{\beta} = 0. \tag{S3}$$

We can now use the Lagrangian multiplier μ to incorporate the constraint into the objective function:

$$L(\boldsymbol{\beta}) = \frac{1}{2}||\mathbf{y} - \mathbf{X}\boldsymbol{\beta}||^2 - \mu\,(||\mathbf{y}||^2 - \mathbf{y}^\top \mathbf{X}\boldsymbol{\beta}).$$

The gradient of L w.r.t to $\boldsymbol{\beta}$ is given by

$$\nabla_{\boldsymbol{\beta}}\, L(\boldsymbol{\beta}) = \mathbf{X}^\top(\mathbf{X}\boldsymbol{\beta} - \mathbf{y}) + \mu \mathbf{X}^\top \mathbf{y}.$$

Setting the gradient to zero, writing $\theta = 1 - \mu$ and solving for $\boldsymbol{\beta}$ yields

$$\boldsymbol{b} = \theta\,(\mathbf{X}^\top\mathbf{X})^{-1}\,\mathbf{X}^\top\mathbf{y} \quad \text{(OLS)}.$$

In other words, the solution is the standard OLS solution $\boldsymbol{\beta}_{\text{ols}} = (\mathbf{X}^\top\mathbf{X})^{-1}\,\mathbf{X}^\top\mathbf{y}$ scaled by the factor θ. Inserting $\boldsymbol{b}$ into Eq. (S3) yields

$$\theta = \frac{||\mathbf{y}||^2}{\mathbf{y}^\top \mathbf{H}\,\mathbf{y}} \tag{S4}$$

with $\mathbf{H} = \mathbf{X}\,(\mathbf{X}^\top\mathbf{X})^{-1}\mathbf{X}^\top$. For OLS, it can be further noted that the denominator is equal to

$$\mathbf{y}^\top\mathbf{H}\,\mathbf{y} = \mathbf{y}^\top\mathbf{H}^\top\,\mathbf{H}\,\mathbf{y} = ||\hat{\mathbf{y}}||^2$$

where $\hat{\mathbf{y}}$ are the predictions under the OLS model and $\mathbf{H} = \mathbf{H}^\top\mathbf{H}$ follows from $\mathbf{H}$'s idempotence and symmetry. For Ridge regression and Kernel Ridge, using a similar derivation we obtain

$$\boldsymbol{b} = \theta\,(\mathbf{X}^\top\mathbf{X} + \lambda\mathbf{I})^{-1}\,\mathbf{X}^\top\mathbf{y} \quad \text{(Ridge)}$$
$$\boldsymbol{b} = \theta\,(\Phi(\mathbf{X})^\top\Phi(\mathbf{X}) + \lambda\mathbf{I})^{-1}\,\Phi(\mathbf{X})^\top\mathbf{y} \quad \text{(Kernel Ridge).}$$

Eq. (S4) still holds, but the hat matrices are given by

$$\begin{aligned}
\mathbf{H} &= \mathbf{X}\,(\mathbf{X}^\top\mathbf{X} + \lambda\mathbf{I})^{-1}\,\mathbf{X}^\top \quad \text{(Ridge)}\\
\mathbf{H} &= \Phi(\mathbf{X})\,(\Phi(\mathbf{X})^\top\Phi(\mathbf{X}) + \lambda\mathbf{I})^{-1}\,\Phi(\mathbf{X})^\top\\
&= \Phi(\mathbf{X})\Phi(\mathbf{X})^\top\,(\Phi(\mathbf{X})\Phi(\mathbf{X})^\top + \lambda\mathbf{I})^{-1}\\
&= \mathbf{K}\,(\mathbf{K} + \lambda\mathbf{I})^{-1} \quad \text{(Kernel Ridge).}
\end{aligned}$$

In all cases, the loss function is convex and the constraint is linear, so the optimization problem is convex. Therefore, the first order stationarity condition is sufficient and the solution represents a unique global minimum.

3 BOUNDED CORRELATION CONSTRAINT

Here, we use the Karush-Kuhn-Tucker (KKT) approach to derive the OLS solution for a bounded correlation constraint. There is two possible scenarios to consider: First, if we have $|\text{corr}(\mathbf{y}, \mathbf{e})| < \rho$ for the unconstrained problem, none of the constraints is active and we are done. Otherwise, we have $\text{corr}(\mathbf{y}, \mathbf{e}) = \rho$ or $\text{corr}(\mathbf{y}, \mathbf{e}) = -\rho$. If we consider $\text{corr}(\mathbf{y}, \mathbf{e}) = \rho$ first, we can expand the correlation term to

$$\frac{\mathbf{y}^\top(\mathbf{y} - \hat{\mathbf{y}})}{||\mathbf{y}||\;||\mathbf{y} - \hat{\mathbf{y}}||} = \rho \tag{S5}$$

which rearranges to

$$||\mathbf{y}||^2 - \mathbf{y}^\top\hat{\mathbf{y}} - \rho\,||\mathbf{y}||\;||\mathbf{y} - \hat{\mathbf{y}}|| = 0. \tag{S6}$$

We can now define the Lagrangian function

$$L(\boldsymbol{\beta}) = \frac{1}{2}||\mathbf{y} - \mathbf{X}\boldsymbol{\beta}||^2 - \mu\,(||\mathbf{y}||^2 - \mathbf{y}^\top\hat{\mathbf{y}} - \rho\,||\mathbf{y}||\;||\mathbf{y} - \hat{\mathbf{y}}||)$$

whose gradient w.r.t to $\boldsymbol{\beta}$ is given by

$$\nabla_{\boldsymbol{\beta}} L(\boldsymbol{\beta}) = \mathbf{X}^\top(\mathbf{X}\boldsymbol{\beta} - \mathbf{y}) + \mu\,\mathbf{X}^\top\mathbf{y} + \mu\rho\frac{||\mathbf{y}||}{||\mathbf{y} - \hat{\mathbf{y}}||}\mathbf{X}^\top\mathbf{y} - \mu\rho\frac{||\mathbf{y}||}{||\mathbf{y} - \hat{\mathbf{y}}||}\mathbf{X}^\top\mathbf{X}\boldsymbol{\beta}.$$

Setting the derivative to zero gives us the solution

$$\boldsymbol{\beta} = (\mathbf{X}^\top\mathbf{X})^{-1}\,\mathbf{X}^\top\mathbf{y}\,(1-\mu-\mu\rho\frac{||\mathbf{y}||}{||\mathbf{y}-\hat{\mathbf{y}}||})(1-\mu\rho\frac{||\mathbf{y}||}{||\mathbf{y}-\hat{\mathbf{y}}||})^{-1}$$

or alternatively

$$\boldsymbol{\beta} = (\mathbf{X}^\top\mathbf{X})^{-1}\,\mathbf{X}^\top\mathbf{y}\,(1+\frac{\mu}{1-\mu\rho\frac{||\mathbf{y}||}{||\mathbf{y}-\hat{\mathbf{y}}||}}).$$

Since the last term is a scalar, the solution, if it exists, is again a scaled version of the OLS solution. The same result is obtained for the case corr$(\mathbf{y},\mathbf{e}) = -\rho$, with a sign reversal for the terms containing ρ. In other words, we are looking for an optimal scaling term $\theta \in \mathbb{R}$. Since the scaling of $\boldsymbol{\beta}$ implies scaling of $\hat{\mathbf{y}}$ by the same amount, we can find the solution by performing a line search and solving Eq. (S6) for θ:

$$||\mathbf{y}||^2 - \mathbf{y}^\top(\theta\,\hat{\mathbf{y}}) - \rho\,||\mathbf{y}||\,||\mathbf{y}-(\theta\,\hat{\mathbf{y}})|| = 0.$$

After squaring both sides and rearranging one obtains

$$\theta^2\,[(\mathbf{y}^\top\hat{\mathbf{y}})^2 - \rho^2||\mathbf{y}||^2||\hat{\mathbf{y}}||^2] - 2\theta||y||^2\,\mathbf{y}^\top\hat{\mathbf{y}}\,(1-\rho^2) + ||y||^4\,(1-\rho)^2 = 0.$$

We can divide by $c := (\mathbf{y}^\top\hat{\mathbf{y}})^2 - \rho^2||\mathbf{y}||^2||\hat{\mathbf{y}}||^2$ and complete the square. This requires that $c \neq 0$ and hence corr$(\mathbf{y},\hat{\mathbf{y}}) \neq \rho^2$, which needs to be verified first. We then arrive at the two solutions

$$\theta_{1,2} = ||\mathbf{y}||^2\,\mathbf{y}^\top\hat{\mathbf{y}}\,(1-\rho^2)/c \pm \frac{||\mathbf{y}||^2}{|c|}\sqrt{\rho^2\,(1-\rho^2)\,(||\mathbf{y}||^2\,||\hat{\mathbf{y}}||^2\,-(\mathbf{y}^\top\hat{\mathbf{y}})^2)}.$$

The roots are always real numbers since $0 \leq \rho^2 \leq 1$ and $||\mathbf{y}||^2\,||\hat{\mathbf{y}}||^2 \geq (\mathbf{y}^\top\hat{\mathbf{y}})^2$. The two solutions for θ define values for which corr$(\mathbf{y},\mathbf{e}) = -\rho$ and corr$(\mathbf{y},\mathbf{e}) = \rho$, respectively.
